# Fatigue in Multiple Sclerosis: A Look at the Role of Poor Sleep

**DOI:** 10.3389/fneur.2015.00021

**Published:** 2015-02-12

**Authors:** Lauren B. Strober

**Affiliations:** ^1^Neuropsychology and Neuroscience Laboratory, Kessler Foundation, West Orange, NJ, USA; ^2^New Jersey Medical School, Rutgers, The State University of New Jersey, Newark, NJ, USA

**Keywords:** fatigue, multiple sclerosis, sleep disorders, depression, fatigue management

## Abstract

Fatigue is a frequent and debilitating symptom of multiple sclerosis (MS) with rates ranging anywhere from 53 to 90%. Despite its high prevalence and grave impact on overall functioning and quality of life, the accurate definition, quantification, and etiology of fatigue have plagued the MS literature and clinical care for decades. With regard to its etiology, MS-related fatigue has been construed as being either primary or secondary. Primary fatigue is purported to be related to centrally mediated processes of the disease whereas secondary fatigue is thought to be a result of the host of factors that may accompany MS (e.g., depression, sleep disturbance). The present paper focuses on secondary fatigue and the role of sleep disturbance, in particular. Despite the intuitive assumption that sleep problems could contribute to fatigue, sleep problems in MS have gone fairly unrecognized until recently. The present paper provides a brief review of the literature pertaining to the prevalence and nature of sleep problems in MS as well as their association with fatigue. A replication of this author’s and others work is presented further demonstrating that sleep disturbance is a significant contributor to fatigue in MS when taking into account disease variables, depression, and sleep disturbance.

## Introduction

Since the first report of fatigue being a prevalent and significant problem in multiple sclerosis (MS) (1) the definition, accurate quantification of fatigue in MS, and etiology has perplexed investigators. To date, nearly every article pertaining to MS-related fatigue contains some sort of disclaimer regarding our inadequate definition, lack of appropriate assessment tools, and limited understanding of its etiology (2). The present article is no exception. However, the investigation outlined in this paper is a replication of previous work demonstrating the fact that when attempting to at least better understand the *etiology* of fatigue in MS, consideration should be given to the role that sleep disturbance as it has been proven to be a significant factor (3).

**Figure 1:**
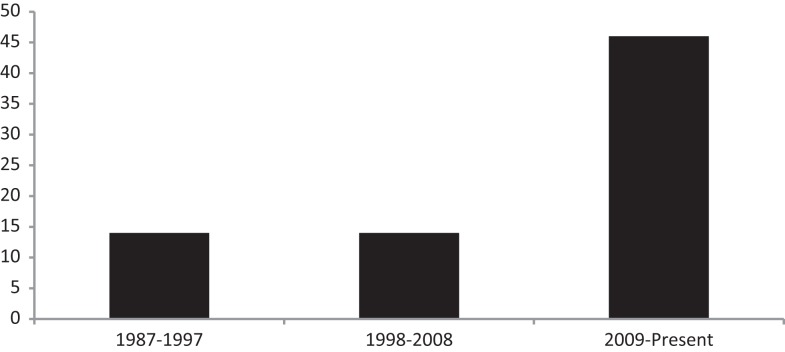
**Number of articles published in PubMed with the words “sleep” and “multiple sclerosis” in the title**.

Despite being the most obvious factor, sleep disturbance or disorders, had initially received fairly little attention as a precipitating or exacerbating factor of fatigue in MS. Fortunately, following an editorial by Attarian titled, “Importance of sleep in the quality of life of multiple sclerosis patients: a long under-recognized issue” sleep disturbance and its disorders have received significantly more attention (4). In fact, when conducting a PubMed search with the terms “MS” and “sleep” in the title, 14 articles have been published between the years 1987 and 1997 and 15 articles were dated from 1998 to 2008, suggesting approximately 15 published articles on sleep in MS per decade. However, since Attarian’s editorial in 2009 the number of published articles with MS and sleep in the title is 46. Thus, at this rate, the number of published articles on sleep in MS over the past 5 years is one a half times more than what was published in the preceding two decades of the 2009 editorial (see Figure 1).

These articles have been published worldwide and span the gamut of looking at the incidence of various sleep disorders to the role of sleep on quality of life and fatigue to the effects of treating sleep problems in MS. While the majority of these studies rely on self-report measurement, some have also included more objective measures including polysomnography. What is to follow is a brief review of fatigue in MS, the prevalence and nature of sleep disorders in MS, and the relationship between the two. Study findings from a recent investigation that replicates previous work demonstrating that sleep disturbance is a significant contributor to fatigue in MS when taking into account disease variables, depression, and sleep disturbance is then presented. Finally, data from a few studies that have demonstrated that effective treatment of such sleep problems can result in reduced fatigue among individuals with MS are provided.

## Background

### Fatigue in MS

Fatigue is a frequent and debilitating symptom of MS with rates ranging anywhere from 53 to 90% (1, 5–9). MS-related fatigue has typically been construed as being either primary or secondary. Primary fatigue is purported to be related to centrally mediated processes of the disease, such as demyelination and axonal loss in the central nervous system or immunological factors. Secondary fatigue, on the other hand, is thought to be a result of the host of factors that may accompany MS (e.g., depression, reduced activity, medication side effects, sleep disturbance) (10). The present paper focuses on secondary fatigue and the role of sleep disturbance. However, regardless of the exact etiology, fatigue is known to be extremely detrimental to those with MS with nearly 55% of patients reporting fatigue to be among their worst symptoms (11) and 40% describing it as their most disabling symptom (8). MS-related fatigue has also been shown to have detrimental effects on daily functioning, social and occupational obligations, and overall well-being. In fact, Freal et al. showed that of the 87% of MS patients complaining of significant fatigue, 67% reported experiencing it on a daily basis and 22% reported that it interfered with their daily functioning (1). Similarly, Iriarte et al. found that fatigue produced limitations in daily functioning in 66% of those who complained of fatigue (12). Of this, 37% reported that it limited their social activities and 61% reported that fatigue limited their work. This is consistent with reports showing that fatigue leads to patients having to cut down on working hours (13) and findings that individuals with MS report fatigue as the one of the greatest culprits related to work difficulty or leaving the workforce (14). Patients also report that their fatigue can result in a lowered sense of self-worth as well as feelings of shame, sorrow, and anger related to their perceptions of their fatigue (15). Similarly, fatigue in MS has been shown to be related to lowered positive affect, psychological distress, and a sense of loss of control (16). Given its grave impact, identification of factors associated with fatigue has been a main priority in research and clinical care among individuals with MS.

### Sleep disorders in MS

Current research suggests that anywhere from 19 to 67% of individuals with MS experience some sort of sleep difficulty (17–32), with rates as high as 80% in some samples (33). Restless leg syndrome (RLS) (27, 28, 30), periodic limb movement (PLM) (21), narcolepsy–cataplexy syndrome, rapid eye movement (REM) behavior disorder, insomnia, and obstructive sleep apnea (OSA) (34) have all been reported (see Table 1).

**Table 1:** **Prevalence of sleep disorders in multiple sclerosis (MS)**.

Reference	Country	Size	Disorder(s)	Prevalence MS	Controls
Bamer et al. (17)	USA	1062	Disturbed sleep^a^	52%	–
Lunde et al. (26, 35)	Norway	90	Poor sleep^b^	67%	44%
Merlino et al. (29)	Italy	120	Poor sleep^b^	48%	–
Chen et al. (19)	China	21	Poor sleep^b^	62%	–
			Initial insomnia	43%	
			Middle insomnia	76%	
			Terminal insomnia	33%	
Pokryszko-Dragan et al. (31)	Poland	100	Initial insomnia	28%	–
			Middle insomnia	33%	
			Terminal insomnia	48%	
Stanton et al. (32)	USA	60	Initial insomnia	42%	–
Braley et al. (33)	USA	30	Obstructive sleep apnea	80%	63%
Kaminska et al. (23)	Canada	62	Obstructive sleep apnea	58%	47%
Dias et al. (20)	USA	103	Obstructive sleep apnea	42%	–
Braley et al. (18)	USA	195	Obstructive sleep apnea	21%	–
Kallweit et al. (22)	Germany	69	Sleep disordered breathing	41%	
Manconi et al. (27)	Italy	861	Restless legs syndrome	19%	4%
Manconi et al. (27)	Italy	82	Restless legs syndrome	37%	–
Moreira et al. (30)	Italy	44	Restless legs syndrome	27%	–
Kaminska et al. (23)	Canada	62	Restless legs syndrome	27%	6%
Kaynak et al. (7, 24)	Turkey	37	Restless legs syndrome	38%	0
Ferini-Strambi et al. (21)	Italy	25	Periodic limb movement	36%	8%
Kaminska et al. (23)	Canada	62	REM sleep behavior	3%	0
Kaminska et al. (23)	Canada	62	Narcolepsy	2%	0

*^a^Based on the medical outcome study sleep (moss) scale*.

*^b^Based on the Pittsburgh sleep quality index*.

In general, it is believed that individuals with MS are three times more likely to experience sleep difficulties than controls (36), though the incidence may be greater for some disorders. For instance, in a large study consisting of 861 individuals with MS and 649 healthy controls, it was found that the risk for RLS was 5.4 times greater for individuals with MS (28).

For many sleep disorders, a direct neurological etiology associated with MS has been found. For instance, cervical lesions have been found to be associated with RLS (27) while greater lesion load in the brainstem and cerebellum has been implicated in PLM (21). Narcolepsy in MS has been suggested to be related to focal lesions in the hypothalamus (37). Finally, REM-behavior sleep disorders in MS have been linked to dorsal pontine lesions (37). While these sleep disorders have a direct etiology related to specifically to MS, insomnia, and OSA, which are also common in the general public have been found to be more prevalent in MS. Insomnia in MS has been attributed to a multitude of factors, primarily nocturia, leg spasms, muscle stiffness, pain, depression, and symptomatic MS medication side effects (32, 38). It has been previously shown that individuals report bladder incontinence as the greatest contributor to disturbed sleep, followed by muscle stiffness and leg spasms, in more than 50% of patients (38). Similar findings were found by Stanton et al. in which nocturia was the most common cause of middle insomnia (72%), followed by pain/discomfort (22%). Nocturia was also the greatest contributor to terminal insomnia (40%), while anxiety/racing mind accounted for initial insomnia most often (28%). In general, depression has been shown to be more related to initial insomnia, while nocturia has been found to be the cause of middle and terminal insomnia (32). With regard to OSA, again, reports of OSA in MS are as high as 80% compared to 63% of healthy controls. Causes of OSA in MS may include inactivity due to disability, brainstem lesions that affect the respiratory centers or nucleus ambiguous, or symptomatic medications that relax muscle tone in the pharynx (37). While these sleep disorders are perhaps the most prevalent in MS, they are both amenable to treatment as will be discussed.

### Relationship of fatigue and sleep in MS

Nearly all studies examining sleep problems in MS have been an attempt to understand its relationship to secondary fatigue and potential mediating effects, with a few exceptions. In particular, a number of studies in the MS literature have focused on sleep disturbance as a significant contributor to fatigue in MS (23, 24, 30, 32, 39–41). For instance, when comparing fatigued and non-fatigued individuals with MS, Kaynak et al. found that those suffering from fatigue experience greater disturbance in sleep microstructure such as total arousal index (TAI), a measure of sleep fragmentation, and PLM index (24). Such findings are consistent with Chen et al.’s findings in which fatigue scores correlated with PLM index, PLM arousal index, REM latency, and TAI (19). Moriera et al.’s study also found that individuals diagnosed with RLS reported poorer sleep quality, which was in turn related to fatigue (30). Finally, reports on a self-report measure of OSA have also been shown to be related to reports of fatigue with more fatigued individuals being more likely to have elevated scores on this measure, even when items that could be construed as fatigue were removed (18, 20).

When constructing a model of fatigue in MS that took into account disease severity, sleep, and depression, it was found that sleep disturbance was the greatest predictor of fatigue, accounting for 24% of the variance followed by depression (10%) and disease severity (9%) (3). It should be noted that this study utilized measures in which overlapping items of fatigue, sleep, and depression were removed from the measures so as to have the purest constructs. These findings were later replicated by Ghajarzadeh et al. in an Iranian sample in which sleep disturbance accounted for 25% of the variance and depression accounted for an additional 9% (42). Such findings suggest that sleep may be the greatest culprit in the experience of fatigue in MS. Within, present findings demonstrate an additional, third replication of these findings in hopes of furthering the increase in attention to sleep problems in MS and their contribution to fatigue in MS.

## Research Design and Methods

### Participants

Participants were recruited from local MS clinics and chapters of the National Multiple Sclerosis Society throughout United States. Participants were enrolled in a larger study examining factors associated with employment in MS and thus were all employed, between the ages of 20 and 64, and diagnosed with definite MS.

### Procedures

Participants completed an online survey consisting of several measures assessing disease variables, psychological functioning, well-being, health–behaviors, adjustment and coping to MS, and overall quality of life. All procedures were approved by the Institutional Review Board of the Kessler Foundation. For the purposes of the present study, participants completed the following measures.

### Measures

#### Modified fatigue impact scale

Modified fatigue impact scale (MFIS) is modified form of the fatigue impact scale (43) that is based on 21 items derived from interviews with MS patients concerning how fatigue impacts their lives. It consists of three subscales: physical, cognitive, and psychosocial functioning. Patients are asked to rate on a scale of 0–4 their agreement with the statement and how it impacts them with “0” being “Never” and “4” being “Almost always.” Recently, it has been recommended that a cutoff of 38 on the MFIS was most indicative of significant fatigue in MS (44). Given the potential overlap of fatigue and depressive symptoms on the psychosocial subscale, the sum of the physical and cognitive subscales was used for the regression analyses. This score has been termed the MFISPC.

#### Pittsburgh sleep quality index

The Pittsburgh sleep quality index (PSQI) is a measure of sleep disturbance and quality (45). It consists of 19 items that are summed to create seven “component” scores: subjective sleep quality, sleep latency, sleep duration, habitual sleep efficiency, sleep disturbances, daytime dysfunction, and use of sleeping medication. The sum of scores for these seven components yields one global score. A cutoff of “5” on the global score is indicative of being a “poor” sleeper. The global score was used in all analyses.

#### Chicago multiscale depression inventory

The Chicago multiscale depression inventory (CMDI) was specifically designed to assess depression in MS and other medical groups (46). It consists of three subscales: evaluative, mood, and vegetative. Each subscale contains 14 items and patients are asked to rate on a scale of 1–5 the extent to which each word/phrase describes them during the past week, including today with “1” being “Not at All” and “5” being “Extremely.” For the purpose of this study, ratings on the vegetative scale were removed due to confounds of sleep and fatigue included in the items on this scale. For our sample, depression was measured by a total score of only the combined evaluative and mood subscales. This score has been termed the CMDIME.

### Statistical analyses

All statistical analyses were conducted using SPSS 21.0 computer software. Pearson correlations were conducted for all correlational analyses. Independent *t*-tests were conducted to examine group differences between the sleep disturbed and non-sleep disturbed groups on demographics, disease variables, fatigue, and depression. Finally, a stepwise, hierarchical regression was conducted with disease duration, sleep disturbance, and depression as the independent variables and fatigue as the dependent variable.

## Results

A total of 107 individuals with definite MS were enrolled in the study. Participants were primarily female (*N* = 92; 86%) with a mean age of 44.71 (±9.76) and mean disease duration of 8.91 (±7.13). Per the global score of the PSQI, 61% of participants were classified as “poor sleepers” and constitute the “sleep disturbed” group. Per the previously recommended cutoff of 38 on the MFIS, approximately half of the sample (47%) experienced significant fatigue (see Table 2).

**Table 2:** **Participant demographics and group comparisons on disease variables, sleep, fatigue, depression**.

	Total sample (*N* = 107) mean (SD)	Range	
Age	44.71 (9.76)	23–64	
Disease duration	8.91 (7.13)	0–31	
PSQI total	7.07 (4.07)	1–18	
MFIS physical	17.47 (8.00)	0–35	
MFIS cognitive	16.00 (9.34)	0–38	
MFIS psychosocial	3.40 (1.92)	0–8	
CMDI mood	22.83 (10.07)	14–68	
CMDI evaluative	19.38 (8.19)	14–61	
CMDI vegetative	33.70 (9.72)	17–53	

	**Not sleep disturbed (*N* = 42) mean (SD)**	**Sleep disturbed (*N* = 65) mean (SD)**	***t*-test, sig**.

Age	46.00 (10.32)	43.88 (9.36)	*t* = 1.10, *p* = 0.274
Disease duration	8.19 (6.07)	9.38 (7.75)	*t* = −0.84, *p* = 0.401
PSQI sleep duration	0.24 (0.43)	0.95 (0.99)	*t* = −5.12, *p* < 0.001
PSQI sleep disturbances	1.12 (0.45)	1.60 (0.49)	*t* = −5.18, *p* < 0.001
PSQI sleep latency	0.50 (0.59)	1.49 (0.89)	*t* = −6.93, *p* < 0.001
PSQI daytime dysfunction	0.74 (0.63)	1.48 (0.77)	*t* = −5.42, *p* < 0.001
PSQI sleep efficiency	0.10 (0.48)	1.11 (1.25)	*t* = −5.88, *p* < 0.001
PSQI sleep quality	0.60 (0.50)	1.65 (0.80)	*t* = −8.39, *p* < 0.001
PSQI sleep medication	0.24 (0.66)	1.20 (1.29)	*t* = −5.08, *p* < 0.001
PSQI global score	3.36 (1.27)	9.48 (3.38)	*t* = −1.50, *p* < 0.001
MFIS physical	14.81 (7.82)	19.18 (7.70)	*t* = −2.84, *p* = 0.006
MFIS cognitive	12.62 (7.47)	18.18 (9.82)	*t* = −3.31, *p* = 0.001
MFIS psychosocial	2.79 (1.88)	3.80 (1.85)	*t* = −2.75, *p* = 0.007
CMDI mood	21.02 (9.85)	24.00 (10.11)	*t* = −1.50, *p* = 0.136
CMDI evaluative	18.26 (8.14)	20.10 (8.20)	*t* = −1.14, *p* = 0.257
CMDI vegetative	28.07 (5.22)	37.34 (10.24)	*t* = −6.16, *p* < 0.001

*PSQI, Pittsburgh sleep quality index; MFIS, modified fatigue impact scale; CMDI, Chicago multiscale depression inventory*.

Initial correlations found physical and cognitive fatigue to be significantly correlated with poor sleep (*r* = 0.42 and 0.49, *p* < 0.001, respectively). A slightly lower correlation was found with psychosocial fatigue (*r* = 0.30, *p* = 0.001). Fatigue was also found to be associated with all of the subscales of the CMDI (*r*’s ranging from 0.24 to 0.72), with the highest correlations occurring with the vegetative subscale (*r*’s ranging from 0.47 to 0.72), which is expected given the overlap of fatigue items on the vegetative scale. Finally, sleep was not found to be related to the mood or evaluative subscales of the CMDI but was significant correlated with the vegetative subscale (*r* = 0.65, *p* < 0.001). Again, this is greatly influenced by item overlap. When looking at the measure scores used in the regression analyses (MFISPC and CMDIME) the correlations between fatigue, depression, and sleep remained (see Table 3).

**Table 3:** **Correlations of fatigue, depression, and sleep disturbance**.

	Mood	Evaluative	Vegetative	Sleep	MFISPC
Physical	0.32**	0.29**	0.50**	0.42**	–
Cognitive	0.33**	0.24*	0.72**	0.49**	–
Psychosocial	0.37**	0.29**	0.47**	0.30**	–
Mood	–	–	–	0.14	–
Evaluative	–	–	–	0.13	–
Vegetative	–	–	–	0.65**	–
MFISPC	–	–	–	0.51**	–
CMDIME	–	–	–	0.14	0.34**

*Physical, MFIS physical subscale; Cognitive, MFIS cognitive subscale; Psychosocial, MFIS psychosocial subscale; Mood, CMDI mood subscale; Evaluative, CMDI evaluative subscale; Vegetative, CMDI vegetative subscale; Sleep, PSQI global score; MFISPC, MFIS Physical + Cognitive score; CMDIME, CMDI Mood + Evaluative score*.

*Significant at the 0.05 level.

**Significant at the 0.01 level.

Stepwise regression analyses found that sleep disturbance accounted for 25% of the variance in predicting fatigue, followed by depression, which accounted for an additional 7%. Together, they accounted for 32% of the variance, while disease duration did not contribute to predicting fatigue (see Table 4).

**Table 4:** **Stepwise hierarchical regression predicting fatigue with disease duration, sleep, and depression as the independent variables**.

	*B*	SE (*B*)	β	*R*^2^
**Step 1**
PSQI	1.95	0.32	0.51**	0.25
**Step 2**
PSQI	1.80	0.31	0.47**	
CMDIME	0.24	0.07	0.28**	0.32

*PSQI, Pittsburgh sleep quality index global score; CMDIME, Chicago multiscale depression inventory mood and evaluative subscales*.

**Significant at the 0.01 level.

Such findings are a near replication of previous findings, suggesting that sleep disturbance is the greatest predictor of fatigue in MS when examined among disease variables and depression, and accounts for a quarter of the variance. However, it should be noted that there are limitations of the present data, including the lack of objective measures of disease severity, fatigue, and sleep disturbance and the use of self-report surveys. There is also the lack of information pertaining to participants’ medication load. Such factors may also contribute to sleep problems in MS. Though, even with the limitations of the present data, clinical findings, and research supporting, the significant role of sleep on MS-related fatigue should hopefully compel practitioners to consider more routine assessment of sleep and referral to sleep studies, when warranted, or when presented with a patient complaining of significant, debilitating fatigue that may exceed what one expects in MS. Consideration should also be given when there is any indication from the patient or bed partner that they patient may have a concomitant sleep disorder. Lunde et al. provide a very thorough review of how best to begin to assess sleep problems in MS as there are presently no specific guidelines. Practitioners are urged to refer to the full guidelines provided by Lunde et al. when treating individuals who complain of significant fatigue, daytime sleepiness/dysfunction, and sleep difficulties. We turn now to a brief discussion pertaining to the findings that proper assessment and treatment of sleep problems in MS may result in reduced fatigue.

### Treating sleep problem in MS

Additional support for addressing sleep problems as an underlying cause of fatigue is more recent findings that effective treatment of sleep problems actually results in reductions in self-reported fatigue and sleepiness in MS. More specifically, in a controlled, non-randomized clinical study, Cote et al. evaluated 62 individuals with MS and referred those suspected of having a sleep disorder for evaluation and treatment at a sleep disorder clinic. Of the 39 (63%) who were diagnosed with a sleep disorder, 21 were treated and 18 were not. Treatment consisted of sleep hygiene advice and then further treatment, which was dependent on the nature of the sleep disorder and included continuous positive airway pressure (CPAP) or other position devices for sleep apnea; treatment of exacerbating factors (e.g., iron or B12 deficiency) and/or pramipexole for RLS; clonazepam for REM behavior disordered sleep; and cognitive behavioral therapy for insomnia. Three months follow-up revealed a significant improvement in fatigue as well as sleepiness, subjective sleep quality, depression, pain, and quality of life among those who were treated. Those not treated did not demonstrate such improvement (39). In another study, progressive muscle relaxation was also shown to improve sleep quality and reports of fatigue in a sample of 32 individuals with MS (47). More specifically, the average score of the fatigue severity scale (48) decreased from 5.75 ± 0.95 (a score above the recommended cutoff of 4) to 3.81 ± 1.30 (*p* < 0.001). Finally, Veauthier et al. evaluated the effectiveness of compliance with treatment imposed by a sleep specialist in 42 individuals with MS. Those who were described as having “good compliance” to the treatment demonstrated a significant, 15-point difference on the MFIS. Such significant findings were not found in those with no to moderate compliance or those without a sleep disorder that were followed over the same time period (40). While it is possible, there may be some expectation bias among those that adhere to their treatment and subsequent ratings of improvement, it should be noted that those who were partially compliant to their treatment also demonstrated an improvement in fatigue, albeit not statistically significant. Together, these findings further stress the importance of proper assessment and effective treatment of sleep problems among individuals with MS complaining of significant fatigue and suggest that effective assessment, referral, and treatment of sleep problems in MS are likely to yield significant results.

In sum, the present paper aimed to again increase our awareness of the prevalence and etiology of sleep problems in MS and more importantly, its contribution to the experience of fatigue, one of the most disabling symptoms associated with MS. The study described within also provides further support of the role of sleep on fatigue and the importance of its assessment. In science, observation is the first step in questioning and aiming to understand a phenomenon. MS-related fatigue has been a construct that has perplexed investigators for decades. Over the past few years, the field has begun to test the hypothesis that sleep may be a significant contributor of fatigue in MS. These observations and subsequent findings have yielded positive results. However, the next, and sometimes neglected aspect of science, is replication. Here, findings show a third replication of study findings that consistently demonstrate that sleep problems account for approximately a quarter of the variance of fatigue in MS.

Based on these findings, further research is warranted in which we continue to examine and model the contributing factors of fatigue and sleep in MS in hopes of indentifying the factors and ultimately, treating them. In doing so, future studies should address some of the methodological limitations of past studies, including reliance on self-report measures of sleep and fatigue. Objective measurement of sleep and fatigue, while time consuming and costly, are likely to yield more substantial findings. A few preliminary studies demonstrating the effects of treating sleep on fatigue were also provided within. Further intervention studies are warranted with larger sample sizes and great characterization of the sleep problems as well as the active ingredients of treatment. With such advances, it is hoped that we will see an increase in the assessment, referral, evaluation, and treatment of sleep problems in MS and ultimately be capable of improving the lives and well-being of individuals with MS.

## Conflict of Interest Statement

The author declares that the research was conducted in the absence of any commercial or financial relationships that could be construed as a potential conflict of interest.

## References

[1] Freal JE Kraft GH Coryell J Symptomatic fatigue in multiple sclerosis. Arch Phys Med Rehabil (1984) 6:135–8. 6703889

[2] Mills RJ Young CA A medical definition of fatigue in multiple sclerosis. Q J Med (2008) 101:49–60 10.1093/qjmed/hcm122 18194977

[3] Strober LB Arnett PA. An examination of four models predicting fatigue in multiple sclerosis (MS). Arch Clin Neuropsychol (2005) 20(5):631–46. 10.1016/j.acn.2005.04.002 15894455

[4] Attarian HP Brown KM Duntley SP Carter JD Cross AH. The relationship of sleep disturbances and fatigue in multiple sclerosis. Arch Neurol (2004) 61:525–8. 10.1001/archneur.61.4.525 15096400

[5] Colosimo C Millefiorini E Grasso MG Vinci F Fiorelli M Koudriavtseva T Fatigue in MS is associated with specific clinical features. Acta Neurol Scand (1995) 92:353–5. 10.1111/j.1600-0404.1995.tb00145.x 8610485

[6] Ford H Trigwell P Johnson M. The nature of fatigue in multiple sclerosis. J Psychosom Res (1998) 45(1):33–8. 10.1016/S0022-3999(98)00004-X 9720853

[7] Kaynak H Altintas A Kaynak D Uyanik O Saip S Agaoglu J Fatigue and sleep disturbance in multiple sclerosis. Eur J Neurol (2006) 13:1333–9. 10.1111/j.1468-1331.2006.01499.x 17116216

[8] Krupp LB Alvarez L LaRocca NG Scheinberg LC Fatigue in multiple sclerosis. Arch Neurol (1988) 45:435–7 10.1001/archneur.1988.00520280085020 3355400

[9] Van der Werf SP Jongen PJH Lycklama a Nijeholt GJ Barkhof F Hommes OR Bleijenberg G. Fatigue in multiple sclerosis: interrelations between fatigue complaints, cerebral MRI abnormalities, and neurological disability. J Neurol Sci (1998) 160:164–70. 10.1016/S0022-510X(98)00251-2 9849800

[10] DeLuca J Fatigue: its definition, its study, and its future. In: DeLuca J, editor. Fatigue as a Window to the Brain. Cambridge, MA: MIT Press (2005). p. 319–25.

[11] Fisk JD Pontefract A Ritvo PG Archibald CJ Murray TJ The impact of fatigue on patients with multiple sclerosis. Can J Neurol Sci (1994) 21:9–14. 8180914

[12] Iriarte J Subira ML de Castro P. Modalities of fatigue in multiple sclerosis: correlation with clinical and biological factors. Mult Scler (2000) 6:124–30. 10.1177/135245850000600212 10773859

[13] Smith MM Arnett PA. Factors related to employment changes in individuals with multiple sclerosis. Mult Scler (2005) 11(5):602–9. 10.1191/1352458505ms1204oa 16193900

[14] Simmons RD Tribe KL McDonald EA. Living with multiple sclerosis: longitudinal changes in employment and the importance of symptom management. J Neurol (2010) 257(6):926–36. 10.1007/s00415-009-5441-7 20084515

[15] Flenser G Ek AC Soderhamn O. Lived experience of MS-related fatigue-a phenomenological interview study. Int J Nurs Stud (2003) 40:707–17. 10.1016/S0020-7489(03)00010-5 12965162

[16] Krupp LB. Fatigue in multiple sclerosis: definitions, pathophysiology, and treatment. CNS Drugs (2003) 17(4):225–34. 10.2165/00023210-200317040-00002 12665396

[17] Bamer AM Johnson KL Kraft GH Amtmann D Sleep problems in multiple sclerosis: assessing prevalence and the association with depression. Mult Scler (2007) 13(S2):S122.

[18] Braley TJ Segal BM Chervin RD. Obstructive sleep apnea and fatigue in patients with multiple sclerosis. J Clin Sleep Med (2014) 10(2):155–62. 10.5664/jcsm.3442 24532998 PMC3899317

[19] Chen J Liu X Sun H Huang Y. Sleep disorders in China: clinical, polysomnography study, and review of the literature. J Clin Neurophysiol (2014) 31(4):375–81. 10.1097/WNP.0000000000000067 25083851

[20] Dias RA Hardin KA Rose H Agius MA Apperson ML Brass SD. Sleepiness, fatigue and risk of obstructive sleep apnea using the STOP-BANG questionnaire in multiple sclerosis: a pilot study. Sleep Breath (2012) 16:1255–65. 10.1007/s11325-011-0642-6 22270686

[21] Ferini-Strambi L Filippi M Martinelli V Oldani A Rovaris M Zucconi M Nocturnal sleep study in multiple sclerosis: correlations with clinical and brain magnetic resonance imaging findings. J Neurol Sci (1994) 125:194–7. 10.1016/0022-510X(94)90035-3 7807167

[22] Kallweit U Baumann CR Harzheim M Hidalgo H Pohlau D Bassetti CL. Fatigue and disordered breathing in multiple sclerosis: a clinically relevant association? Mult Scler Int (2013) 2013:1–7. 10.1155/2013/286581 24251039 PMC3819751

[23] Kaminska M Kimoff RJ Benedetti A Robinson A Bar-Or A Lapierre Y Obstructive sleep apnea is associated with fatigue in multiple sclerosis. Mult Scler (2012) 18(8):1159–69 10.1177/1352458511432328 22183937

[24] Kaynak H Altintas A Kaynak D Uyanik O Saip S Agaoglu J Fatigue and sleep disturbance in multiple sclerosis. Eur J Neurol (2006) 13:1333–9. 10.1111/j.1468-1331.2006.01499.x 17116216

[25] Lobentanz IS Asenbaum S Vass K Sauter C Klosch G Kollegger H Factors influencing quality of life in multiple sclerosis patients: disability, depressive mood, fatigue, and sleep quality. Acta Neurol Scand (2004) 110:6–13. 10.1111/j.1600-0404.2004.00257.x 15180801

[26] Lunde HMB Aae TF Indrevag W Aarseth J Bjorvatn B Myhr K Poor sleep in patients with multiple sclerosis. PLoS One (2012) 7(11):e49996 10.371/journal.pone.0049996 23166808 PMC3498191

[27] Manconi M Rocca MA Ferini-Strambi L Tortorella P Agosta F Comi G Restless leg syndrome is a common finding in multiple sclerosis and correlates with cervical cord damage. Mult Scler (2008) 14:86–93. 10.1177/1352458507080734 17942519

[28] Italian REMS Study Group Manconi M Ferini-Strambi L Filippi M Bonanni E Iudice A Multicenter case-control study on restless legs syndrome in multiple sclerosis: the REMS study. Sleep (2008) 31(7):944–52. 18655317 PMC2491510

[29] Merlino G Fratticci L Lenchig C Valente M Cargnelutti D Picello M Prevalence of ‘poor sleep’ among patients with multiple sclerosis: an independent predictor of mental and physical status. Sleep Med (2009) 10:26–34. 10.1016/j.sleep.2007.11.004 18207453

[30] Moreira NC Damasceno RS Medeiros CA Bruin PF Teixeira CA Horta WG Restless leg syndrome, sleep quality and fatigue in multiple sclerosis patients. Braz J Med Biol Res (2008) 41:932–7. 10.1590/S0100-879X2008001000017 19030714

[31] Pokryszko-Dragan A Bilinska M Gruszka E Biel L Kaminska K Konieczna K Sleep disturbances in patients with multiple sclerosis. Neurol Sci (2012) 34(8):1291–6 10.1007/s10072-012-1229-0 23109097 PMC3747317

[32] Stanton BR Barnes F Silber E. Sleep and fatigue in multiple sclerosis. Mult Scler (2006) 12:481–6. 10.1191/135248506ms1320oa 16900762

[33] Braley TJ Chervin RD Segal BM. Fatigue, tiredness, lack of energy, and sleepiness in multiple sclerosis patients referred for clinical polysomnography. Mult Scler Int (2012) 2012:1–7. 10.1155/2012/673936 23316361 PMC3539354

[34] Fleming WE Pollak CP Sleep disorders in multiple sclerosis. Semin Neurol (2005) 25:64–8 10.1055/s-2005-867075 15798938

[35] Lunde HMB Bjorvatn B Myhr KM Bo L Clinical assessment and management of sleep disorders in multiple sclerosis: a literature review. Acta Neurol Scand (2012) 127(Suppl 196):24–30 10.1111/ane.12046 23190288

[36] Clark CM Fleming JA Li D Oger J Klonoff H Paty D. Sleep disturbance, depression, and lesion site in patients with multiple sclerosis. Arch Neurol (1992) 49:641–3. 10.1001/archneur.1992.00530300077013 1596200

[37] Brass SD Duquette P Prouix-Therrien J Auerbach S Sleep disorders in patients with multiple sclerosis. Sleep Med Rev (2010) 14:121–9 10.1016/j.smrv.2009.07.005 19879170

[38] Strober LB Arnett PA Sleep changes in multiple sclerosis (MS): from the individual’s perspective. J Sleep Disord Treat Care (2013) 2(4). 10.4172/2325-9639.1000122

[39] Côté I Trojan D Kaminska M Cardoso M Benedetti A Weiss D Impact of sleep disorder treatment on fatigue in multiple sclerosis. Mult Scler (2013) 19(4):480–9. 10.1177/1352458512455958 22914848

[40] Veauthier CM Gaede G Radbruch H Gottschalk S Wernecke D Paul F. Treatment of sleep disorders may improve fatigue in multiple sclerosis. Clin Neurol Neurosurg (2013) 115:1826–30. 10.1016/j.clineuro.2013.05.018 23764040

[41] Veauthier C Radbruch H Gaede G Pfueller CF Dorr J. Fatigue in multiple sclerosis is closely related to sleep disorders: a polysomnographic cross-sectional study. Mult Scler (2011) 17(5):613–22. 10.1177/1352458510393772 21278050

[42] Ghajarzadeh M Sahraian MA Fateh R Daneshmand A. Fatigue, depression, and sleep disturbances in Iranian patients with multiple sclerosis. Acta Med Iran (2012) 50(4):244–9. 22592574

[43] Fisk JD Ritvo PG Ross L Haase DA Marrie TJ Schlech WF. Measuring the functional impact of fatigue: initial validation of the fatigue impact scale. Clin Infect Dis (1994) 18(Suppl1):S79–83. 10.1093/clinids/18.Supplement_1.S79 8148458

[44] Flachenecker P Kumpfel T Kallmann B Gottschalk M Grauer O Rieckmann P Fatigue in multiple sclerosis: a comparison of different rating scales and correlation to clinical parameters. Mult Scler (2002) 8:523–6. 10.1191/1352458502ms839oa 12474995

[45] Buysse DJ Reynolds CF Monk TH Berman SR Kupfer DJ. The Pittsburgh sleep quality index (PSQI): a new instrument for psychiatric research and practice. Psychiatry Res (1989) 28(2):193–213. 10.1016/0165-1781(89)90047-4 2748771

[46] Nyenhuis DL Luchetta T Yamamoto C Terrien A Bernardin L Rao SM The development, standardization, and initial validation of the Chicago multiscale depression inventory. J Pers Assess (1998) 70(2):386–401. 10.1207/s15327752jpa7002_14 9697337

[47] Dayapogu N Tan M. Evaluation of the effect of progressive relaxation exercises on fatigue and sleep quality in patients with multiple sclerosis. J Altern Complement Med (2012) 18(10):983–7. 10.1089/acm.2011.0390 22967281 PMC3469207

[48] Krupp LB LaRocca NG Muir-Nash G Steinberg G. The fatigue severity scale. Application to patients with multiple sclerosis and systemic lupus erythematosus. Arch Neurol (1989) 46:1121–3. 10.1001/archneur.1989.00520460115022 2803071

